# AKR1C enzymes sustain therapy resistance in paediatric T-ALL

**DOI:** 10.1038/s41416-018-0014-0

**Published:** 2018-03-08

**Authors:** Roberta Bortolozzi, Silvia Bresolin, Elena Rampazzo, Maddalena Paganin, Francesca Maule, Elena Mariotto, Daniele Boso, Sonia Minuzzo, Valentina Agnusdei, Giampietro Viola, Geertruy te Kronnie, Giovanni Cazzaniga, Giuseppe Basso, Luca Persano

**Affiliations:** 1 Department of Women’s and Children’s Health, University of Padova, Padova, 35128 Italy; 2 Istituto di Ricerca paediatrica Città della Speranza–IRP, corso Stati Uniti 4, Padova, 35127 Italy; 3 Clinic of paediatric Oncohematology, University Hospital of Padova, Padova, 35128 Italy; 4 Department of Surgery, Oncology and Gastroenterology, University of Padova, Padova, 35128 Italy; 5 Istituto Oncologico Veneto - IRCCS, Padova, 35128 Italy; 6 Department of paediatric, Centro Ricerca M. Tettamanti, University of Milano Bicocca, Fondazione MBBM, Monza, 20900 Italy

**Keywords:** Cancer therapeutic resistance, Acute lymphocytic leukaemia

## Abstract

**Background:**

Despite chemotherapy intensification, a subgroup of high-risk paediatric T-cell acute lymphoblastic leukemia (T-ALL) patients still experience treatment failure. In this context, we hypothesised that therapy resistance in T-ALL might involve aldo-keto reductase 1C (AKR1C) enzymes as previously reported for solid tumors.

**Methods:**

Expression of NRF2-AKR1C signaling components has been analysed in paediatric T-ALL samples endowed with different treatment outcomes as well as in patient-derived xenografts of T-ALL. The effects of AKR1C enzyme modulation has been investigated in T-ALL cell lines and primary cultures by combining AKR1C inhibition, overexpression, and gene silencing approaches.

**Results:**

We show that T-ALL cells overexpress AKR1C1-3 enzymes in therapy-resistant patients. We report that AKR1C1-3 enzymes play a role in the response to vincristine (VCR) treatment, also ex vivo in patient-derived xenografts. Moreover, we demonstrate that the modulation of AKR1C1-3 levels is sufficient to sensitise T-ALL cells to VCR. Finally, we show that T-ALL chemotherapeutics induce overactivation of AKR1C enzymes independent of therapy resistance, thus establishing a potential resistance loop during T-ALL combination treatment.

**Conclusions:**

Here, we demonstrate that expression and activity of AKR1C enzymes correlate with response to chemotherapeutics in T-ALL, posing AKR1C1-3 as potential targets for combination treatments during T-ALL therapy.

## Introduction

The nuclear factor erythroid 2 (NF-E2)-related factor 2 (NRF2)–Kelch-like (ECH)-associated protein 1 (KEAP1) signaling pathway is a cellular system that protects cells from oxidative stress and insults from toxic xenobiotics.^1^ Indeed, the NRF2 axis is finely regulated in normal cells by acting as a surveillance system to prevent the accumulation of endogenous metabolites or hazardous substrates from the microenvironment. Nonetheless, in the past decade, multiple studies reported a significant overactivation of this pathway in many tumors suggesting a positive correlation between its enhanced activity and poor prognosis in cancer patients.^1,2^ Under basal conditions, NRF2 activity is repressed by binding to its inhibitor KEAP1, which leads to NRF2 proteasomal degradation through the CUL3-dependent ubiquitin ligase complex.^3^ Oxidation of KEAP1 mediated by oxidative stress or electrophilic agents induces the release of NRF2 and its translocation into the nucleus where it binds to ARE consensus sequences and transactivates a series of cytoprotective target genes, including aldo-keto reductase (AKR) family members.^4^

The superfamily of AKR enzymes catalyze the NADPH-dependent conversion of aldehydes and ketones to their corresponding alcohols. Thus, a wide variety of substrates that include simple carbohydrates, cellular metabolites, steroid hormones, endogenous prostaglandins, xenobiotic compounds, and chemotherapeutics are potential targets of these enzymes.^5^ The AKR1C subfamily includes four isoenzymes (AKR1C1-4) widely expressed in different human tissues, except for AKR1C4 that is liver-specific.^6^ Recent studies demonstrated that AKR1C1-3 are highly expressed in many human cancers including prostate,^7^ breast,^4^ glioma,^8,9^ neuroblastoma,^10^ lung,^11,12^ and acute myeloid leukemia (AML), where they mediate drug resistance, regulate cell differentiation, and promote cancer cell proliferation.^13,14^

In this context, it has been demonstrated that the pan AKR1C inhibitor medroxyprogesterone acetate (MPA), greatly enhances the anti-leukemic activity of bezafibrate, by inhibiting the prostaglandin D_2_ 11b-ketoreductase activity of AKR1C enzymes, thus promoting growth arrest, apoptosis, and cell differentiation in AML cells.^15^ Moreover, different studies aimed to selectively inhibit specific AKR1C isoforms (i.e., AKR1C3) and revealed that inhibition of AKR1C3 alone is not adequate to exert an anti-leukemic effect in AML cells,^16^ thus reinforcing the hypothesis that redundant activity of AKR1C enzymes supports intracellular antioxidant response.

In acute lymphoblastic leukemia (ALL), AKR1C1-4 messenger RNA (mRNA) and protein expression has been correlated with cellular sensitivity to the mustard pro-drug PR-104A,^17^ the latter was reported to be converted and activated by AKR1C3 in a subset of cancer cell lines.^18^ Although Moradi Manesh et al.^17^ nicely demonstrated that AKR1C3 expression and enzymatic activity are more abundant in T-ALL compared to BCP-ALL xenografts, and positively correlate with the response to the mustard pro-drug PR-104A, little is known about the potential correlation between AKR1C1-3 expression and the response to standard treatment regimens in ALLs.

Recent improvements in chemotherapeutic protocols for childhood ALL achieve a 5-year survival rate of about 80%.^19^ However, different studies identified a slower clearance of cancer cells during treatment in T-ALL compared to B-ALL,^20^ with more resistant tumors (high-risk T-ALL) showing a 7-year survival of only 40%.^19^ Therefore, the study of the mechanisms underlying drug response and the development of new therapeutic strategies for patients who poorly respond to current treatment protocols remains an important challenge in T-ALL. In this context, we hypothesised that AKR1C enzymes could perturb therapeutic success in T-lineage leukemia. We evaluated the expression and enzymatic activity of AKR1Cs in a large cohort of T-ALLs, finding a significant upregulation of AKR1C1-3 in “resistant” or “poorly responding” tumors. Moreover, we functionally validated the role exerted by these enzymes in controlling T-ALL cell response to chemotherapeutics by pharmacologically or genetically modulating their activity. Finally, we directly correlated the expression of AKR1C1-3 to chemotherapy response in patient-derived xenograft (PDX)-T-ALL samples.

## Materials and Methods

### Primary leukemia cell cultures

T-cell acute lymphoblastic leukemia cells derived from bone marrow (BM) of patients were obtained after informed consent following the tenets of the Declaration of Helsinki and according to the guidelines of the ethics committee of the University of Padova, the Padova Academic Hospital, and the Italian Association of paediatrics Onco-Hematology (AIEOP). Diagnosis was obtained according to standard cytomorphology, cytochemistry, and immunophenotypic criteria.^21^ All analysed T-ALL samples were obtained after hemolysis of red blood cells at the time of diagnosis, before treatment. Derived T-ALL cells have then been subjected to total RNA and/or protein extraction according to standard procedures or were used for drug testing and injected in mice to generate PDX-T-ALL as described hereafter. T-ALL patients have been classified as therapy “responders” or “resistant” according to the minimal residual disease (MRD) molecularly detected at day 78 from the start of therapy.

In some experiments, primary and PDX-T-ALL cells were seeded at a concentration of 10^5^ cells per well in 96-well microtiter plates and cultured in α-MEM medium supplemented with 10% FBS, 1% penicillin/streptomycin, 1% glutamine (all from Thermo Fisher Scientific, Waltham, MA), 10% human heat inactivated AB+ serum (Sigma-Aldrich S.r.l., Milan, Italy), human IL7 (20 ng/ml; R&D Systems, Minneapolis, MN), human Stem Cell Factor (SCF) (50 ng/ml), human FLT3-ligand (20 ng/ml; both from Peprotech, London, UK), and insulin (20 nM; Sigma-Aldrich S.r.l.). Cells were immediately exposed to the test compounds and cell survival was evaluated by MTT assay after 48 h. Clinical information of T-ALL patients, from whom cells included in this study derived, are provided as Suppl. Table S1.

### Measurement of AKR1C enzymatic activity

Bone marrow or peripheral blood derived T-ALL blasts were obtained from cryopreserved samples from our collection. AKR1C enzymatic activity was measured as previously described.^22,23^ Briefly, cells were added to white 96-well plates at 2 × 10^5^ cells per well in phenol red-free media and equilibrated at 37 °C in a 5% CO_2_ incubator for 1 h in the presence of coumberone (10 µM). Coumberol-derived fluorescence intensity was detected after 1–3 h of coumberone addition with a VICTOR3™ Multilabel Plate Reader (excitation: 385 nm; emission: 510 nm; Perkin Elmer, Waltham, MA) and normalised to cell density. The slopes of linear enzymatic reactions obtained by regression analyses have been considered as good surrogates for measuring enzymatic activity and thus reported in the manuscript graphs.

### Generation of T-ALL xenografts

To establish xenografts, 6- to 9-week-old mice were injected intravenously (i.v.) with 10^7^ T-ALL cells in 300 μl of Dulbecco’s phosphate buffered saline as previously described.^24^ NOD/SCID mice were purchased from Charles River (Wilmington, MA). Procedures involving animals and their care conformed to institutional guidelines that comply with national and international laws and policies (EEC Council Directive 86/609, OJ L 358, 12 December 1987) and were authorised by the local ethical committee. T-cell acute lymphoblastic leukemia cell engraftment was monitored by periodic blood drawings and flow cytometry analysis of CD5 and CD7 markers over a 5-month period or until clear leukemia initiation. T-cell acute lymphoblastic leukemia xenograft cells have been derived from engrafted mice spleens and then used for ex vivo experiments. Information regarding T-ALL patients from whom xenografts have been generated are summarised in Suppl. Table S1.

### Combined drug analysis

Leukemia cell lines and primary cultures were treated with cytarabine (Aractyn; AraC), daunorubicin (Dauno), vincristine (VCR) (all from Pfizer, New York, NY) or l-asparaginase (Asp) (Sigma-Aldrich S.r.l.) in the presence or absence of MPA (Sigma-Aldrich S.r.l.), added to each drug solution at fixed combination ratios. Cell viability was determined after 48–72 h of treatment by MTT assay as described above. To determine the synergistic, additive, or antagonistic effects of drug combinations, we used CompuSyn software (ComboSyn Inc., Paramus, NJ; www.combosyn.com) based on the method of the combination index (CI) described by Chou.^25^ Synergy, additivity, and antagonism were defined by a CI <1, CI = 1, or CI >1, respectively. Where indicated for some experiments, T-ALL cell lines have been treated with tert-buthylhydroxyquinone (t-BHQ; Sigma-Aldrich S.r.l.) for 18 h at a final concentration of 5 μM.

## RESULTS

### AKR1C1-3 are overexpressed in T-ALL cells from therapy-resistant patients

**Fig. 1:**
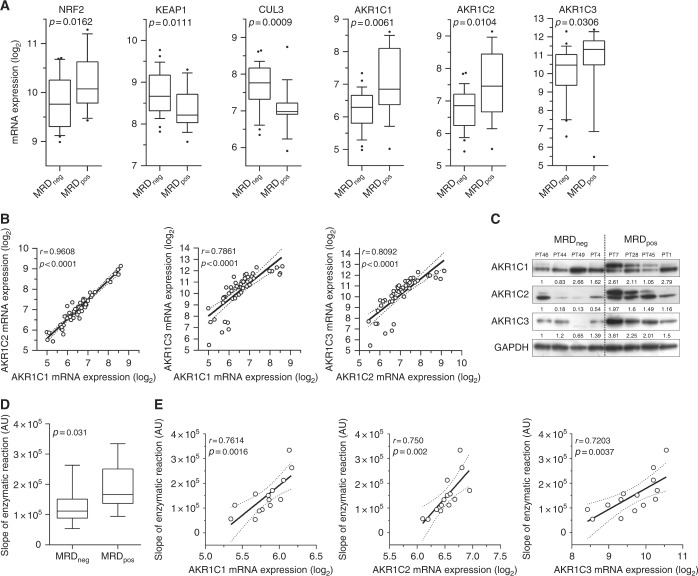
*AKR1C1-3* are overexpressed/activated in T-ALLs from therapy-resistant patients. **a** Box plots showing expression of selected transcripts (as indicated) in MRD_neg_ (*n* = 29) and MRD_pos_ (*n* = 19) T-ALL samples at diagnosis. **b** Graphs reporting the linear correlation existing between the expression values of the single AKR1C isoenzymes. Correlation between the mRNA expression of *AKR1C1* vs. *AKR1C2* (left panel), *AKR1C1* vs. *AKR1C3* (middle panel), and *AKR1C2* vs. *AKR1C3* (right panel) are shown. **c** Western blot analysis of AKR1C1-3 protein expression in MRD subgroups. Relative densitometric values of bands normalised to GAPDH expression are reported below each protein analysed. **d**, **e** Box plot summarising the slope of linear coumberone conversion during time of T-ALL cells from MRD_neg_ (*n* = 11) and MRD_pos_ (*n* = 9) patients **d** and relative correlation with the mRNA expression of each AKR1C isoenzymes (AKR1C1-3) (*n* = 14). mRNA expression of *AKR1C1* (left panel), *AKR1C2* (middle panel), and *AKR1C3* (right panel) vs. the calculated slope of linear coumberone conversion reactions are shown. **e** In correlation graphs, 95% confidence interval is indicated by dotted lines. Moreover, Pearson *r* and relative *p* values are reported

In order to evaluate a potential role of AKR1C1-4 on the phenomenon of therapy resistance in T-ALL, we analysed the expression levels, at diagnosis, of a series of NRF2-AKR1C axis components including *NRF2*, *KEAP1*, *CUL3*, and *AKR1C1-4* in 48 patients for which the transcriptional profile was generated (Suppl. Table S1). T-cell acute lymphoblastic leukemia samples were divided into two subgroups based on their response to first-line treatment (details in Suppl. Materials and Methods). In particular, T-ALL patients have been defined as therapy “responders” when showing absent MRD (MRD_neg_), molecularly detected at day +78 from induction therapy, or “resistant” if MRD >5 × 10^−4^ (MRD_pos_) was detected at the same time point. Indeed, evaluation of MRD levels allows the identification of disease persistence and is considered a powerful predictor of therapy response.^19,20^ We found that MRD_pos_ patients display a significant higher expression of both *NRF2* and *AKR1C1-3* transcripts, together with a lower expression of the *NRF2* inhibitors *KEAP1* and *CUL3* (Fig. 1a). As expected,^6^ *AKR1C4* displayed low/absent expression in T-ALL samples (Suppl. Fig. S1). The expression of *AKR1C* isoenzymes (1–3) was significantly correlated (Fig. 1b). We then confirmed protein expression of AKR1C1-3 in a smaller set of MRD_pos_ vs. MRD_neg_ patients, showing that AKR1C1-3 proteins are overexpressed in MRD_pos_ patient samples (Fig. 1c). Moreover, as a further validation, we measured the speed of the specific conversion of the ketone coumberone metabolite to coumberol (slope of linear enzymatic reaction) as a reliable surrogate of AKR1C1-3 enzyme activity.^22,23^ We found that AKR1C1-3 enzymatic function is enhanced in resistant MRD_pos_ compared to MRD_neg_ T-ALLs (Fig. 1d). Finally, to better characterise the extent of AKR1C1-3 activation in resistant T-ALL samples, we correlated the enzymatic activity of AKR1C isoenzymes (1–3) to their cognate mRNA expression levels obtained for the same patients, showing a highly concordance (Pearson *r* > 0.7) (Fig. 1e). These data support our hypothesis that AKR1C1-3 enzymes could modulate drug sensitivity in T-ALL cells as demonstrated by a clear-cut overexpression and activation in MRD_pos_ T-ALL patients.

### Inhibition of AKR1Cs sensitise T-ALL cell lines to vincristine treatment

**Fig. 2:**
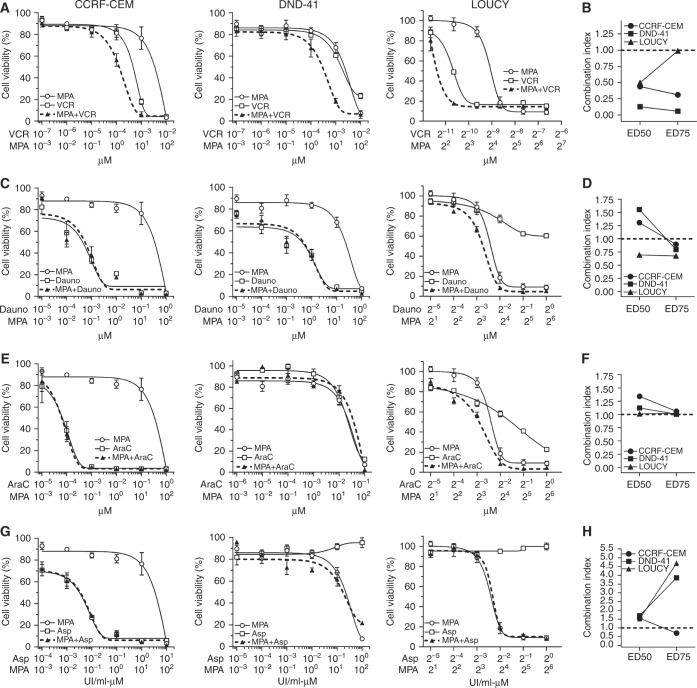
Pan inhibition of AKR1C1-3 enzymes sensitises T-ALL cell lines to vincristine. Dose–response curves of MPA and its combination at constant molar ratio with vincristine (VCR; **a**), daunorubicin (Dauno; **c**), cytarabine (AraC; **e**), and l-asparaginase (Asp; **g**) in T-ALL cell lines. Cell viability was determined by MTT assay after 72 h of drug exposure. Data are expressed as mean ± S.E.M. of at least three independent experiments. Combination index (CI) values were calculated for each drug combination at effective dose (ED) 50 and ED75, respectively (**b**, **d**, **f**, and **h**)

**Fig. 3:**
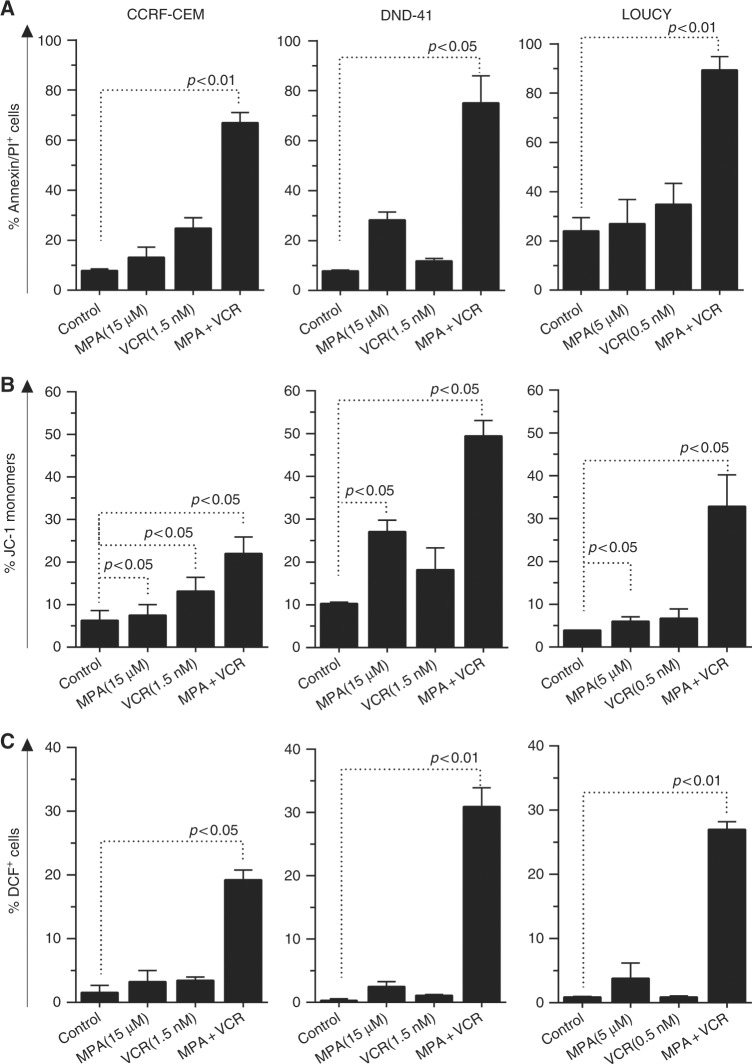
The combined MPA/VCR treatment increases apoptosis in T-ALL cell lines. **a** Analysis of apoptosis (by Annexin-V/PI staining) induced by MPA, VCR, and their combination (at same molar ratios as in Fig. 2) at the indicated concentrations 72 h post treatment. In particular, representative drug dosages shown in graphs have been selected in order to achieve a lethal effect of VCR/MPA combination around 75%. We considered as apoptotic/dead cells all cells being alternatively stained for Annexin-V, PI, or both. **b** Assessment of mitochondrial membrane potential after treatments. Cells were treated with the indicated concentration of compounds for 72 h, then stained with the fluorescent probe JC-1. **c** Evaluation of ROS production after treatments. Cells were treated with the indicated concentration of compound for 72 h and then stained with H_2_-DCFDA. All data are expressed as mean ± S.E.M. of at least three independent experiments. Statistical analysis was assessed by one-way ANOVA with Newman–Keuls multiple comparison post-test

In order to demonstrate the involvement of AKR1C1-3 in the phenomenon of drug resistance, we evaluated if AKR1C1-3 inhibition could enhance drug response in terms of cell viability. To this end, we used a panel of three T-ALL cell lines (CCRF-CEM, DND-41, and LOUCY, all expressing varied levels of AKR1C1-3 isoforms; Suppl. Fig S2A) that were treated with vincristine (VCR), l-asparaginase (ASP), daunorubicin (Dauno), and cytarabine (AraC), all compounds employed during T-ALL therapy,^19^ in combination with MPA, a pan AKR1C inhibitor.^15,26^ As expected, inhibition of AKR1C1-3 by MPA dramatically reduced AKR1C-dependent enzymatic activity as assessed by measurement of coumberone conversion (Suppl. Fig. S2B). Moreover, MPA administration was sufficient to sensitise all T-ALL cell lines examined for VCR treatment, displaying a calculated CI <1, and thus a synergistic action according to the Chou method^25^ (Fig. 2a, b, Suppl. Table S2, and Suppl. Fig. S2C–E). On the contrary, we did not observe any significant increase in efficacy of Dauno, AraC, and ASP treatments when combined with MPA (Fig. 2c–h and Suppl. Table S2). Interestingly, treatment with MPA alone demonstrated a strong reduction of cell viability of CCRF-CEM and DND-41 when used at very high concentrations (i.e., 100 µM), and showed efficacy to only some extent in LOUCY T-ALL cells, even if synergistic potential with VCR was maintained (Fig. 2a–h, Suppl. Table S2, and Suppl. Fig. S2E). We then further characterised the synergistic effect mediated by MPA/VCR combination by analyzing the mechanism of cell death induction after treatment with sub-lethal doses of the single drugs. Indeed, only the combination of both drugs (MPA + VCR) was able to induce a potent pro-apoptotic response in T-ALL cells, which showed a significant increase of apoptotic cells (by Annexin-V/PI staining) after treatment (Fig. 3a, Suppl. Fig. S3A, and Suppl. Fig. S4A).

Since VCR treatment induces cell death via a mitochondrial pathway, thus generating a concurrent production of Reactive Oxygen Species (ROS),^27^ we evaluated if the combination with MPA produced a significant increase of mitochondrial membrane depolarisation (as measured by JC-1 probe) and ROS accumulation (as measured by DCF^+^ cells). VCR treatment, combined with MPA, induced a strong imbalance of mitochondrial potential (Fig. 3b, Suppl Fig. S3B, and Suppl. Fig. S4B) and a significant increase of cellular ROS content (Fig. 3c, Suppl. Fig. S3C, and Suppl. Fig. S4C), supporting the hypothesis that inhibition of the AKR1C1-3-dependent detoxifying mechanism may enhance the anti-cancer effect exerted by some chemotherapics (in particular of VCR).

### Inhibition of AKR1C1-3 sensitise primary T-ALL cells to VCR treatment

**Fig. 4:**
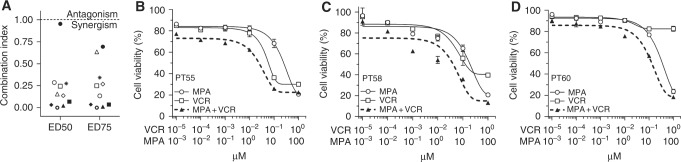
MPA sensitise primary T-ALL cells to vincristine. **a** CI values calculated at ED50 and ED75 for the combination of MPA and VCR in 10 primary cell lines. Cell viability was evaluated by MTT assay after 48 h of treatment with VCR and MPA. **b**–**d** Representative complete dose–response curves of MPA and its combination with VCR for PT55, PT58, and PT60, respectively

To further validate the sensitisation effect exerted by AKR1C1-3 inhibition on VCR treatment, we treated 10 primary T-ALL cultures (derived from leukemia patients at diagnosis) with VCR alone or in combination with MPA. All primary cells tested exhibited a strong synergistic effect mediated by MPA/VCR combination, independently from their response to therapy at day 78, as shown by CI calculation in Fig. 4a (exact CI calculated at ED50 and ED75 and MRD status for each primary T-ALL culture are reported in Suppl. Table S3). Representative complete dose–response curve are shown for PT55, PT58, and PT60 (Fig. 4b–d).

### Modulation of AKR1C1-3 expression regulates T-ALL response to VCR treatment

**Fig. 5:**
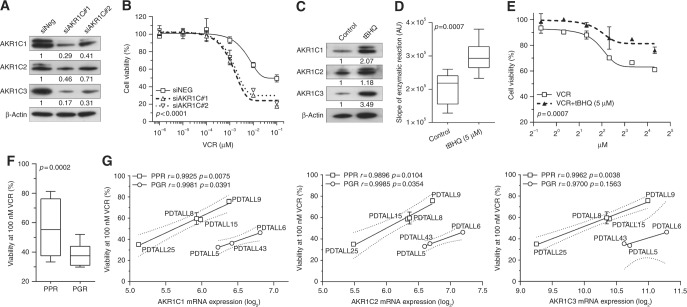
AKR1C1-3 levels affect response to vincristine in T-ALL cells. **a**,** b** After 48 h from electroporation with two different siRNAs against AKR1C1-3, CCRF-CEM cell lysates were analysed by immunoblotting with AKR1C1, AKR1C2, AKR1C3, and β-Actin-specific antibodies, showing the effective gene silencing of AKR1C enzymes **a**. Response of AKR1C-silenced CCRF-CEM cells to scalar doses of VCR (48 h) is reported **b**. **c**, **d** AKR1C1-3 activity was stimulated in CCRF-CEM by t-BHQ treatment (5 μM) for 18 h and then evaluated by WB **c** and calculation of the slope of linear coumberone conversion reaction **d**. **e** Dose–response curves of t-BHQ-stimulated and control CCRF-CEM exposed to scalar doses of VCR for 48 h. **f** Box plot showing the ex vivo response to VCR treatment in terms of cell viability of cells obtained from PPR (*n* = 4) and PGR (*N* = 3) T-ALL patient-derived xenografts (PDX-T-ALLs). **g** Correlation of AKR1C1-3 mRNA expression with the viability of PDTALL cells after ex vivo treatment with 100 nM VCR for 48 h is reported. 95% confidence interval is indicated by dotted lines. All data are expressed as mean ± S.E.M. of three independent experiments. In correlation graphs, Pearson *r* and relative *p* values are reported independently for PGR and PPR patient-derived PDX-T-ALLs

In order to verify if the enhanced anti-cancer effects observed after combination treatment with MPA/VCR is dependent on the inhibition of a specific AKR1C enzyme or either by their combined knockdown due to a shared redundant function, we transfected CCRF-CEM with specific siRNAs against each AKR1C (1–3) isoenzyme or obtained the combined gene silencing of all the three AKR1C isoforms by using two different siRNAs that consistently reduced all their protein expression in T-ALL cells, respectively (Fig. 5a and Suppl. Fig. S5A–C, left panels). Supporting the hypothesised redundancy of AKR1C1-3 enzymes^16^ and confirming the effects obtained with MPA treatment (Fig. 2a), only the combined silencing of all the *AKR1C* genes (C1, C2, and C3) strongly sensitised CCRF-CEM to VCR treatment (Fig. 5b and Suppl. Fig. S5A–C, right panels), however without directly affecting cell proliferation or viability (Suppl. Fig. S5D,E).

In a complementary way, we treated CCRF-CEM with the redox-cycling agent t-BHQ, known to induce transcriptional activation of NRF2, subsequent ARE-driven gene expression and consequent antioxidant protection.^4^ A short treatment of T-ALL cells with t-BHQ was sufficient to induce the overexpression of the three AKR1C isoenzymes (Fig. 5c) and a significant increase of their enzymatic activity assessed by coumberone conversion (Fig. 5d). In agreement with previous results, activation of AKR1C1-3 enzymes significantly protected CCRF-CEM from VCR treatment (Fig. 5e). Together, these data demonstrate that modulation of AKR1C1-3 might play a fundamental role in the mechanism of T-ALL response to VCR treatment.

### AKR1C1-3 expression is directly correlated to VCR resistance ex vivo of T-ALL xenografts

In order to functionally correlate the overexpression of AKR1C1-3 enzymes with the response to VCR also in T-ALL primary cells, we generated seven T-ALL xenografts (PDX-T-ALL) by injecting patient-derived cells into NOD/SCID mice as described.^24^ Grafted T-ALL cells were subjected to gene expression profiling in order to characterize the transcriptional levels of AKR1C1-4. As previously shown for T-ALL primary samples (Suppl. Fig. S1), also T-ALL-derived xenografts showed absent expression of *AKR1C4* gene (data not shown). Moreover, confirming previous results, the mRNA levels of the single AKR1C isoenzymes were strictly correlated (Suppl. Fig. S6).

It has been previously suggested that the outcome of T-ALL patients could partially predict the response of PDXs to different chemotherapics, including VCR, both in vivo and ex vivo.^28^ Based on this suggestion, we decided to correlate *AKR1C1-3* expression and VCR response separately in the group of PDX-T-ALLs generated from prednisone good responder (PGR) or prednisone poor responder (PPR, commonly stratified as high-risk) patients. As expected, PGR patients-derived PDX-T-ALLs displayed a significant higher ex vivo sensitivity to VCR treatment than PPR xenografts (Fig. 5f). In addition, both PDX-T-ALL subgroups displayed a direct correlation between *AKR1C1-3* expression levels and the amount of surviving cells after VCR treatment (Fig. 5g), with the only exception for AKR1C3 correlation in PGR xenografts (Fig. 5g, right panel). Although based on a small number of samples, these results suggest the presence of a direct link between AKR1C1-3 activity and VCR resistance in T-ALL.

### T-ALL induction chemotherapy promotes activation of AKR1C1-3 and resistance to VCR

**Fig. 6:**
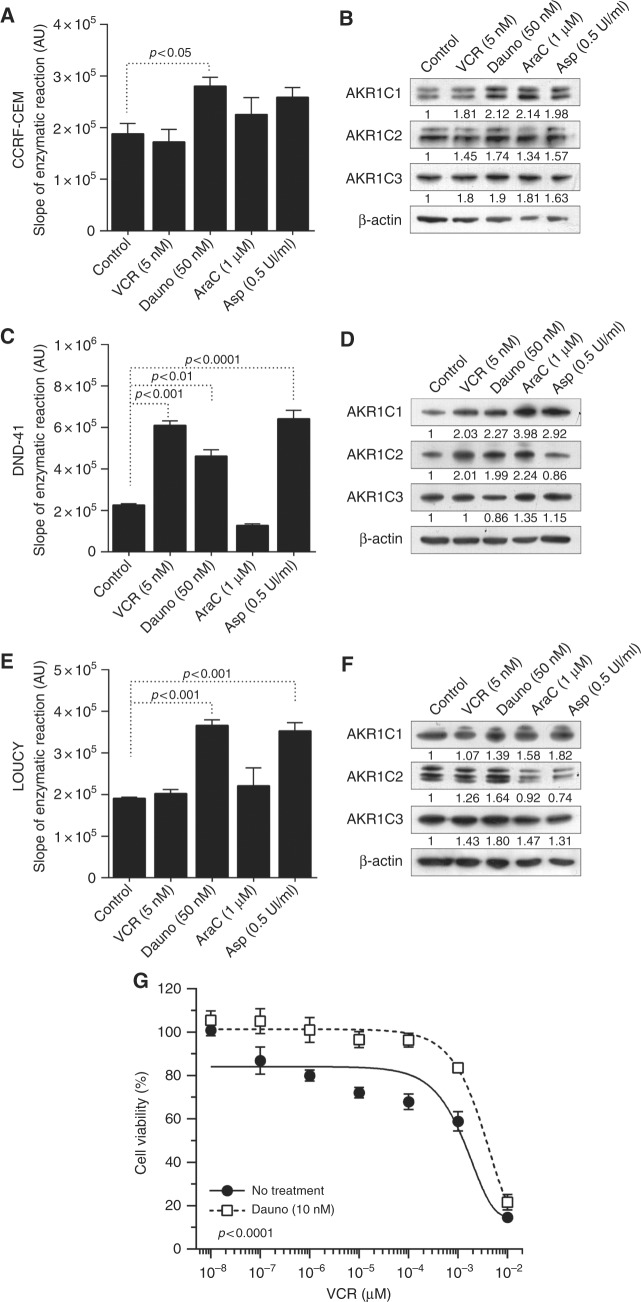
T-ALL induction therapy increases AKR1C1-3 activity/expression in T-ALL cell lines. CCRF-CEM **a**, **b**, DND-41 **c**, **d**, and Loucy **e**, **f** cells were treated with VCR, Dauno, AraC, and Asp at the indicated doses for 24 h. After treatment, AKR1C1-3 activity was determined by calculating the slopes of the linear reaction of coumberol formation from non-fluorescent coumberone **a**, **c**, **d** and relative AKR1C1-3 protein expression determined by WB **b**, **d**, **f**. **g** Dose–response curves of Dauno-pre-treated (10 nM for 24 h) and control DND-41 cells exposed to scalar doses of VCR for 48 h. Data are expressed as mean ± S.E.M. of at least three independent experiments. Statistical analysis was assessed by one-way ANOVA with Newman–Keuls multiple comparison post-test

It has been recently demonstrated that cancer cells can transcriptionally regulate NRF2 and, consequently, AKR1C1-3 activity as a pro-survival response against drug treatments.^4^ Thus, in order to evaluate the potential establishment of such a resistance loop in our experimental setting, we measured the amount of coumberone conversion rate in all T-ALL cell lines after 24 h of treatment with ASP, VCR, Ara-C, and Dauno at sub-lethal concentrations. Only Dauno induced a significant increase in the production of fluorescent coumberol in all T-ALL cell lines. Instead, VCR treatment increased coumberone conversion rate only in DND-41 cells and Asp significantly augmented AKR1C-dependent enzymatic activity in both DND-41 and LOUCY cell lines. Ara-C did not induce any increase of AKR1C1-3 activity (Fig. 6a, c, e). We confirmed these results by Western Blot (WB), showing that Dauno-induced overexpression of AKR1C1-3 proteins in all the three T-ALL cell lines tested (Fig. 6b, d, f). In order to functionally validate these data, we pretreated DND-41 cells with Dauno, with consequent AKR1C1-3 enzymes overexpression and activation (Fig. 6c, d), and tested the response to VCR treatment. Indeed, a 24 h pretreatment of T-ALL cells with Dauno was sufficient to make these cells significantly more resistant to VCR treatment (Fig. 6g). These data corroborate the “open” hypothesis that treatment with certain drugs can influence the response to other agents by activating a NRF2-dependent surveillance system and establish a potential AKR1C-mediated drug resistance loop during T-ALL therapy and highlight the relevance of potential pharmacological inhibition of AKR1C1-3 as adjuvant treatment to current chemotherapy protocols.

## Discussion

Despite of significant improvements in intensive combination chemotherapy and hematopoietic stem cell transplantation achieved in the last decades,^19,29^ a number of childhood T-ALL patients only partially respond to treatment, thus experiencing relapse and poor disease outcome.^19,30^ In this context, AKR1C1-3, being part of the NRF2-KEAP1 signaling pathway,^4^ are intimately linked with cancer biology and could participate in sustaining resistance to anti-cancer treatments.^2,31^ Given their intrinsic promiscuity of substrates,^32^ AKR1C enzymes have been extensively reported to increase cancer cell resistance to therapeutics in various human cancers, by reducing the intracellular levels of drug products, adducts, or compounds themselves. In this study, we investigated if AKR1C1-3 could contribute to therapy resistance in T-ALL by evaluating the potential impact of *AKR1C1-3* expression and inhibition on drug sensitivity in T-ALL cell lines, primary cultures, and PDXs.

MRD levels molecularly detected after induction therapy (day 78) are a good marker of treatment response and predictive of subsequent relapse of T-ALL patients.^19^ For this reason, we chose to analyse NRF2-AKR1C1-3 signaling activation in MRD_neg_ vs. MRD_pos_ (MRD >5 × 10^−4^) T-ALL patients at diagnosis as a reliable and representative distinction of therapy “sensitive” or “resistant” tumors, respectively. Our data clearly show that therapy-resistant T-ALL samples are endowed with a significant overexpression and/or activation of three *AKR1C* (1–3) isoenzymes, with augmented enzymatic activity being dependent on increased gene transcription (Fig. 1).

Exploring the relationship between NRF2 overactivation and drug resistance in cancer, Wang et al.^33^ previously demonstrated that the transient knockdown of NRF2, or its specific inhibition by KEAP1 overexpression, both strongly increased the susceptibility of lung cancer cells to different chemotherapics, including *cis*platin, doxorubicin, and etoposide. Along this line, in our study, we demonstrate that inhibition of AKR1C1-3 function by the pan-AKR1C inhibitor MPA (Figs. 2, 3, and 4) or AKR1C-specific gene silencing (Fig. 5a, b) are sufficient to increase T-ALL cell sensitivity to VCR. This sensitisation effect was obtained by the specific inhibition of AKR1C1-3, without the need to counteract additional detoxifying genes, generally regulated by NRF2, thus indicating a major role of AKR1C family members in modulating drug response in T-ALL. In order to further sustain the direct involvement of AKR1C1-3 enzymes in chemotherapy resistance, we compared the mRNA expression of additional NRF2 targets, previously involved in drug metabolism, such as the glutamate-cysteine ligase catalytic subunit (GCLC), the glutamate-cysteine ligase modifier subunit (GCLM), or the UDP-glucuronosyltransferase 1A6^34,35^ in MRD_pos_ vs. MRD_neg_ T-ALL patients. Gene expression data disclosed that the above mentioned genes were not overexpressed in MRD_pos_ relative to MRD_neg_ patients, thus questioning a potential role in sustaining therapy resistance in T-ALL (data not shown). In line with our results, AKR1C1-3 were reported to induce resistance to *cis*platin in colon cancer^36^ and to repress the sensitivity to ATRA-induced differentiation^37^ or bezafibrate^26^ in AML.

As an additional result, we were able to directly correlate the expression of AKR1C enzymes with the degree of resistance to VCR ex vivo treatment of PDX-T-ALL cells (Fig. 5f, g). In particular, minimal changes in the balance of AKR1C expression were associated to a very different drug efficacy in terms of surviving cells after VCR treatment. This suggests that any little imbalance in the regulation of the AKR1C surveillance system may produce dramatic effects on cellular metabolism, especially during the onset or progression of resistant tumors. Intriguingly, our data clearly show that AKR1C1-3 enzyme levels mainly affect VCR response rather than other chemotherapeutics. Supporting these results, Rovini et al.^38^ previously reported that in parallel with their effects on the microtubule network, vinca alkaloids are able to directly interact with mitochondria, thus inducing an early collapse of mitochondrial potential, with a significant increase of ROS production and apoptosis. In the same conditions, DAUNO or AraC have been demonstrated not to directly affect mitochondrial stability.^39^ In this context, AKR1C enzymes may function as a scavenger of the VCR-dependent mitochondrial depolarisation, by directly counteracting ROS induced-cell damage and subsequent cell death. On the contrary, thanks to their structure, other drugs could be subjected to AKR1C reductase activity, nevertheless producing further toxic metabolites.^40^

AKR1C1-3 expression has been reported to be induced by anti-cancer agents, thus producing a consequent enhancement of the resistance mechanisms engaged by cancer cells against standard chemotherapics and the activation of survival pathways, thus facilitating cancer progression. In particular, Wang et al.^4^ showed that different anti-cancer drugs displayed disparate degrees of NRF2 activation and AKR1C overexpression in A549 lung carcinoma cells, including drugs with no effect (i.e., methotrexate), a modest (i.e., *cis*platin), or strong effect (i.e., BCNU).

Further exploring the connection between AKR1C1-3 and the phenomenon of drug resistance, we found that drugs (Dauno, VCR, and Asp) used as first-line treatment during induction therapy in paediatric T-ALL induced overexpression/activation of AKR1C1-3 (Fig. 6a–f). We established a treatment resistance loop to VCR in surviving cancer cells. Further, we reported that Daunorubicin induced overexpression/activation of AKR1C1-3 in all the three T-ALL cell lines tested (Fig. 6a–f), showing various degree of activation probably depending on the redox status (and thus GSH content) of each particular cell line, as previously suggested.^4^ Moreover, we showed that Dauno-induced AKR1C1-3 overexpression partially counteracted the effects exerted by VCR treatment (Fig. 6g), suggesting the potential antagonistic effect of combined AKR1C-inducing drugs.

From a pharmacological point of view, the availability of novel AKR1C-selective inhibitors would be of value for multiple reasons including: (i) cancer chemoprevention; (ii) potential adjuvant therapies in combination with standard chemotherapics; (iii) direct single therapeutic interventions.^41^ In this context, given the involvement of AKR1C3 in steroid hormone metabolism,^5^ several laboratories performed structural studies in order to identify new potential inhibitors of AKR1C3 for the treatment of hormone-dependent cancers such as prostate or breast carcinomas.^42–44^ However, many previous studies support the notion that a pan-AKR1C inhibition is fundamental to exert an anti-cancer activity^15,41,45^ and, given the structural diversity of these inhibitors such as NCI-PI,^16^ MPA,^15^ and jasmonates,^37^ it is reasonable to conceive that the shared anti-leukemic action of these drugs can be attributed to their ability to inhibit AKR1C isoforms rather than to shared “off-target” effects. In line, pyrithione-based ruthenium complexes have been successfully synthesised, demonstrating a potent inhibition of AKR1C1-3 and cytotoxic effects in breast cancer cells.^46^

In conclusion, our results for the first time directly correlate the expression and activation of AKR1C enzymes to chemotherapy response in paediatric T-ALL, making AKR1C1-3 potential suitable targets for T-ALL therapy or either predictive markers of patient response to treatments at diagnosis. However, increasing efforts are needed in order to better characterize the genetic or epigenetic mechanisms underlying NRF2/AKR1C1-3 de-regulation in T-ALL before they could be exploited as reliable prognostic tools.

From a therapeutic point of view, the future development of new AKR1C inhibitors, with possibly less side effects than MPA, that could be used in combination with the standard chemotherapeutics, may finally promote a more effective therapy response in T-ALL patients.

## Electronic supplementary material

Supplementary data

Supplementary Table S1
