# Supplementary material for: AKR1C enzymes sustain therapy resistance in paediatric T-ALL
Source: Br J Cancer. 2018 Mar 8;118(7):985–94. doi: 10.1038/s41416-018-0014-0 (PMC5931104; doi:10.1038/s41416-018-0014-0)
Supplement: Supplementary file 1 — Supplementary data [file 41416_2018_14_MOESM1_ESM.pdf]

SUPPLEMENTARY INFORMATION

AKR1C enzymes sustain therapy resistance in pediatric T-ALL.

Supplementary data contain additional Materials and Methods, six supplementary figures (S1-S6) and two supplementary tables (S2-S3). Supplementary Table S1 is provided separately as excel file.

SUPPLEMENTARY MATERIALS AND METHODS

Cell cultures and growth inhibition assay.

The human T-leukemia cell lines CCRF-CEM, DND-41 and Loucy were purchased from the American Type Culture Collection (ATCC; Manassas, VI) in 2014. Cells were cultured in RPMI 1640 supplemented with 10% fetal bovine serum (FBS), glutamine (2mM), penicillin (100U/ml) and streptomycin (100µg/ml) (all from Thermo Fisher Scientific, Waltham, MA), and maintained at 37°C in a humidified atmosphere with 5% CO_2. Cells were routinely tested for *Mycoplasma* contamination every 4 months by Venor®GeM Mycoplasma PCR Detection Kit (Minerva Biolabs GmbH, Berlin, Germany).

The cytotoxic activity of selected drugs was determined using a standard 3-[4,5-dimethylthiazol-2-yl]-2,5-diphenyltetrazodium bromide (MTT)-based colorimetric assay (Sigma-Aldrich S.r.l., Milan, Italy). Briefly, cells were seeded at a density of $2.5x10^4$ cells/well in 96-well microtiter plates. After 24 hours (h), cells were exposed to the test compounds. Cell survival was determined by the addition of an MTT solution at different time points (48-72h). In all graphs, MTT assay data have been expressed as relative viability of compound-treated cells normalized to control cells exposed at matched concentrations of drug diluent alone. In particular, treatment of cells with increasing concentrations of drug diluent induced only negligible and not significant variations (<5%) of their viability.

First line therapy of pediatric T-ALL patients.

During the first 78 days of therapy, T-ALL patients have been subjected to a treatment schedule (AIEOP-ALL 2000 protocol; identification #NCT00613457) comprising an "induction" phase from

day 1 to day 33 (daily glucocorticoids, four dosages of Vincristine, four dosages of Daunorubicin and eight dosages of L-asparaginase) and a subsequent "consolidation" phase from day 34 to day 78 (one 28-day cycle of 6-mercaptopurine, two dosages of Cyclophosphamide and four 4-day cycles of Cytarabine) (Schrappe *et al*, 2011).

RNA extraction and gene expression profiling of T-ALL samples.

Total RNA was extracted from T-ALL samples and patient-derived xenografts by TRIzol reagent according to manufacturer's instructions (Thermo Fisher Scientific, Waltham, MA). For microarray experiments, *in vitro* transcription, hybridization and biotin labeling of RNA from T-ALL cells were performed according to Affymetrix 3'IVT Express Kit protocol. GeneChip Human Genome U133 Plus 2.0 (Affymetrix, Santa Clara, CA) was used. Ten out of 48 T-ALL samples were part of a previously published cohort (GSE13159; (Haferlach *et al*, 2010)).

Microarray data (CEL files) were generated using default Affymetrix microarray analysis parameters (Command Console suite software, Affymetrix, Santa Clara, CA). CEL files were normalized using the robust multiarray averaging (RMA) expression measure of Affy-R package (www.bioconductor.org).

After normalization, batch effects were removed between the two cohorts using the ComBat method (Johnson *et al*, 2007). Expression data have been deposited into the Gene Expression Omnibus (GEO) database under Series Accession Number GSE87865 and are accessible without restrictions.

Western Blot Analysis.

Total protein extracts were isolated in lysis buffer as previously described (Milani *et al*, 2014) and protein concentration determined by BCA protein assay (Thermo Scientific Pierce, Waltham, MA). Equal amounts of proteins (10–20μg) were resolved using NativePAGE, Novex Bis-Tris Gels (Thermo Fisher Scientific, Waltham, MA) and transferred to PVDF Immobilon-P Membrane (Merck Millipore, Darmstadt, Germany). Membranes were blocked with I-block (Thermo Scientific Pierce, Waltham, MA) for at least 2h, under rotation at room temperature. Membranes were then incubated overnight at 4°C under constant shaking with the following primary antibodies: AKR1C1 (rabbit, 1:1000), AKR1C2 (mouse, 1:1000, both from Abcam, Cambridge, UK), AKR1C3 (rabbit, 1:1000, Thermo Fisher Scientific, Waltham, MA), and β-actin (mouse, 1:10000, Sigma-Aldrich S.r.l., Milan, Italy) or GAPDH (mouse, 1:1000, GeneTex, Irvine, CA) as loading controls. Membranes were next incubated with HRP-Labeled goat anti-rabbit or anti-mouse IgG (both 1:50000 in I-block; Perkin Elmer, Waltham, MA) for 60min. All membranes were visualized using ECL Select and exposed to Hyperfilm

MP (both from GE Healthcare, Catania, Italy). Densitometric analyses were performed by using ImageJ image analysis software and normalized to their relative loading controls (GAPDH or β-Actin).

Transfection of T-ALL cells.

To achieve a suitable gene silencing, CCRF-CEM cells were transfected with 150pmol of two different small interfering RNAs (siAKR1C#1: 5'-GGAGUAAAUUGCUAGAUUUTT-3' and siAKR1C#2: 5'-CCCUAAUUAUCCAUAUUCATT-3') able to knockdown the expression of AKR1C1-3 enzyme isoforms as well as with a non targeting siRNA (siNEG) (all from Qiagen, Hilden, Germany) using the 4D-Nucleofector™ System with Cell Line 4D-Nucleofector™ Solution SF (Lonza, Basel, Switzerland). Effectiveness of gene silencing was evaluated by Western Blot after 48h from transfection; AKR1C-silenced cells (24h from transfection) were used for testing their response to VCR treatment by MTT assay.

Cytofluorimetric assays.

Cell death was determined by flow cytometry analysis of cells stained for annexin-V-FITC and propidium iodide following the manufacturer's instructions (Annexin-V Fluos, Roche Diagnostics S.p.A., Monza, Italy).

The mitochondrial membrane potential was measured with the lipophilic cation 5,5',6,6'-tetrachlo-1,1',3,3'-tetraethylbenzimidazolyl-carbocyanine (JC-1) (Thermo Scientific Pierce, Waltham, MA) and ROS production was detected by the 2,7-dichlorodihydrofluorescein diacetate (H_2-DCFDA) dye, which is oxidized to the fluorescent compound dichlorofluorescein (DCF) by a variety of peroxides as previously described (Romagnoli *et al*, 2010).

All acquisitions were performed with a Coulter Cytomics FC500 (Beckman Coulter, Brea, CA) flow cytometer.

Statistical analyses

Graphs and associated statistical analyses were generated using Graph Pad Prism 6.07 (GraphPad, La Jolla, CA). All data in bar graphs are presented as mean ± standard error of the mean (S.E.M.). Statistical significance was measured by one-way ANOVA with Newman–Keuls multiple comparison post test (for more than two comparisons) and Mann-Whitney test (comparison of two groups); actual p values have been reported in graphs for each significant difference obtained between analyzed experimental groups.

Correlation between indicated variables has been estimated by calculating Pearson correlation coefficient (r) and considered significant when $p<0.05$.

Statistical differences among the different dose-response curves reported in the manuscript were calculated by interpolating non-linear sigmoidal regressions of each dataset and comparing top, bottom, LogIC50 and Hillslope parameters with Extra sum-of-squares F test.

SUPPLEMENTARY TABLES S2-S3

Supplementary Table S2. Combination Index values calculated for MPA/VCR, MPA/Dauno, MPA/AraC and MPA/Asp combined treatment in T-ALL cell lines.

Cell line	*Drug*	*ED50*	*ED75*
**CCRF-CEM**	VCR	0.44	0.311
	Dauno	1.306	0.897
	AraC	1.341	1.06
	Asp	1.55	0.705
**DND-41**	VCR	0.128	0.059
	Dauno	1.555	0.796
	AraC	1.122	1.003
	Asp	1.7	3.86
**LOUCY**	VCR	0.499	0.989
	Dauno	0.694	0.674
	AraC	1.013	1.019
	Asp	1.52	4.69

ED: Effective Dose.

Supplementary Table S3. Combination Index values calculated for MPA/VCR combined treatment in primary T-ALL cultures.

Sample ID	*MRD status*	*ED50*	*ED75*
**PT55**	NEG	0,136	0,268
**PT56**	N.D.	10^{-5}	$7{,}7x10^{-16}$
**PT57**	POS	0,954	0,694
**PT58**	NEG	0,286	0,134
**PT59**	NEG	0,069	0,036
**PT60**	NEG	0,274	0,342
**PT61**	POS	0,023	0,011
**PT62**	NEG	0,035	0,035
**PT63**	NEG	0,155	0,635
**PT64**	POS	0,244	0,249

ED: Effective Dose. N.D.: Not Determined

SUPPLEMENTARY FIGURES S1-S6

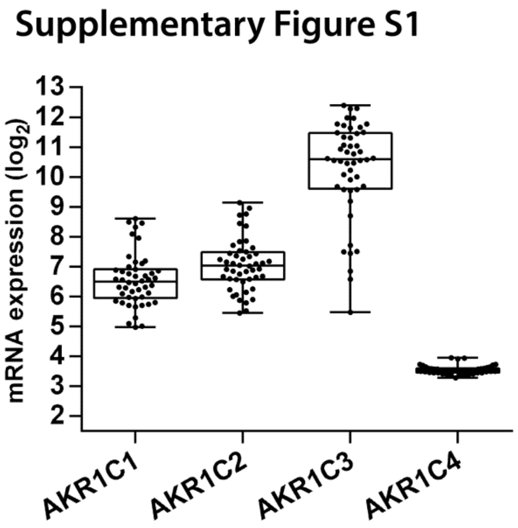

Supplementary Figure S1. Expression of *AKR1C* enzymes in T-ALL samples. Box plot summarizing mRNA expression (log2) of *AKR1C1-4* enzymes in 48 T-ALL samples. Individual samples are reported as black dots.

Supplementary Figure S2

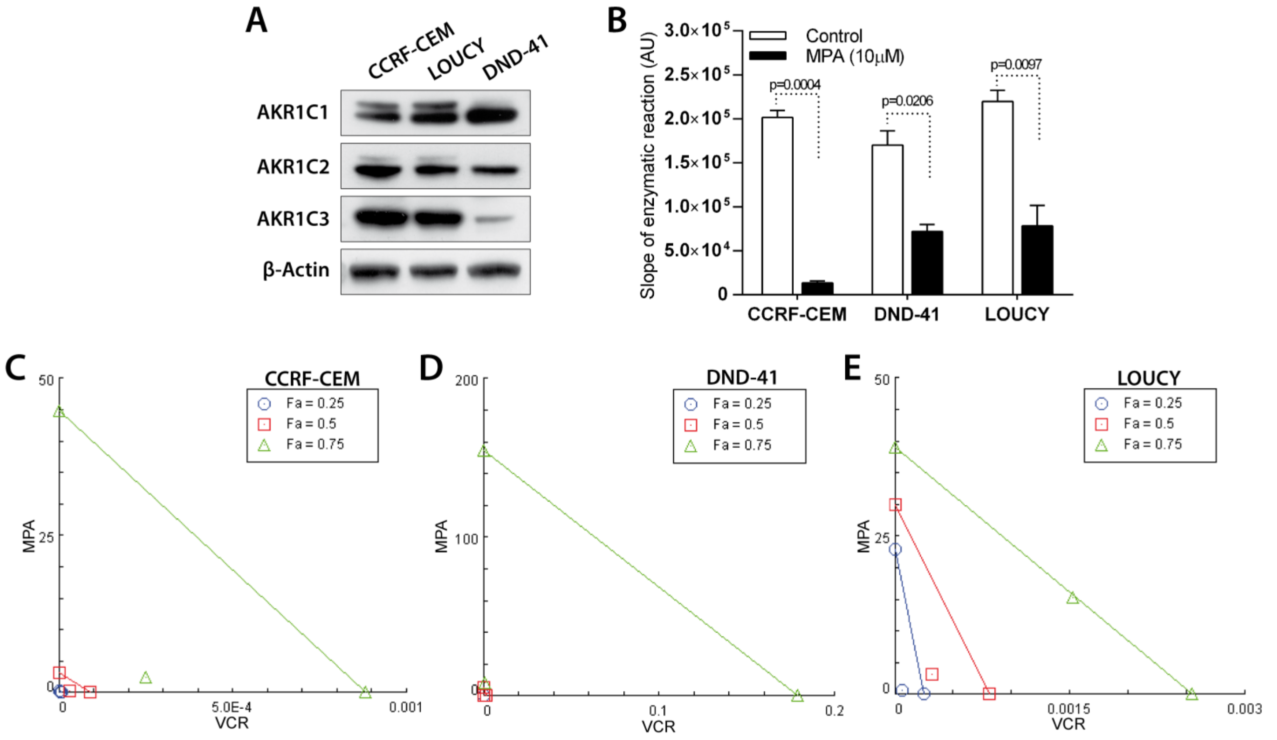

Supplementary Figure S2. MPA treatment abrogates AKR1C1-3-dependent coumberone conversion activity and synergize with VCR in T-ALL cell lines. (**A**) Western blot analysis of AKR1C1-3 and β-Actin protein expression in CCRF-CEM, DND-41 and LOUCY T-ALL cell lines. (**B**) Bar graph confirming the MPA ability to efficiently suppress the AKR1C-dependent enzymatic activity in T-ALL cell lines. Data are expressed as mean ± S.E.M. of at least three independent experiments. Statistical analysis was assessed by t-test. (**C-E**) Isobolograms showing synergism between MPA and VCR in CCRF-CEM (**C**), DND-41 (**D**) and Loucy (**E**) T-ALL cell lines at selected effective doses (Fa). Isolated dots represents the effect of MPA/VCR drug combination which is below the additivity line, thus confirming synergism between compounds.

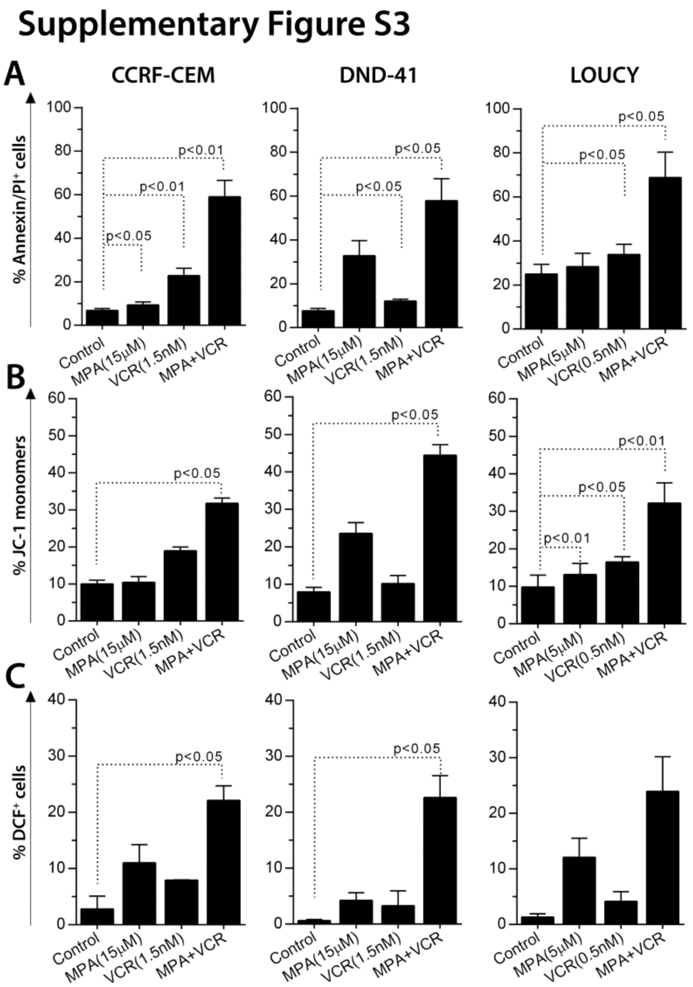

Supplementary Figure S3. The combined MPA/VCR treatment increases apoptosis in T-ALL cell lines through the mitochondrial pathway. (**A**) Analysis of apoptosis induced by MPA, VCR and their combination at the indicated concentrations (48h post treatment). Apoptosis was evaluated by flow cytometry by staining the T-ALL cells with Annexin-V-FITC and propidium iodide. We considered as apoptotic/dead cells all cells being alternatively stained for Annexin-V, PI or both. (**B**) Assessment of mitochondrial membrane potential after treatment of T-ALL cell lines with MPA, VCR and their combination. Cells were treated with the indicated concentration of compounds for 48h, then stained with the fluorescent probe JC-1 for analysis of mitochondrial potential depolarization. Cells were then analyzed by flow cytometry as described in the experimental procedures. (**C**) Evaluation of ROS production after treatment of T-ALL cell lines with MPA, VCR and their combination. Cells were treated with the indicated concentration of compound for 48h and then stained with H2-DCFDA for evaluation of ROS levels by flow cytometry. All data are expressed as mean ± S.E.M. of at least three independent experiments. Statistical analysis was assessed by one-way ANOVA with Newman-Keuls multiple comparison post test.

Supplementary Figure S4

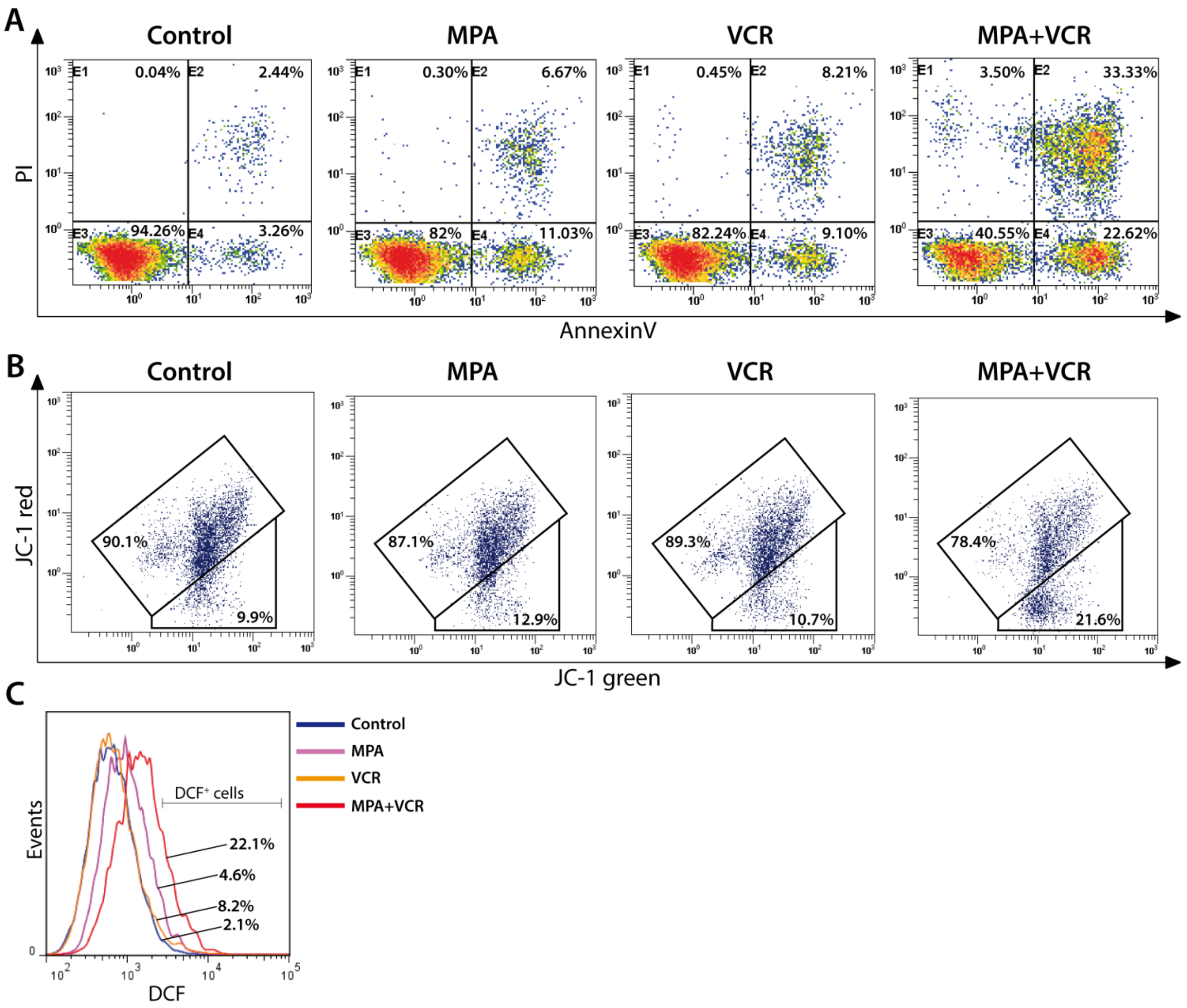

Supplementary Figure S4. Representative cytofluorimetric analyses of apoptosis, mitochondrial membrane depolarization and ROS accumulation in CCRF-CEM cells. Representative dot plots showing Annexin-V/PI (**A**) or JC-1 (**B**) staining of CCRF-CEM cells treated with MPA, VCR or the combination of both and compared to control cells. (**C**) Overlay panel showing a representative experiment of ROS accumulation after MPA+VCR treatment of CCRF-CEM cells compared to the other conditions reported.

Supplementary Figure S5

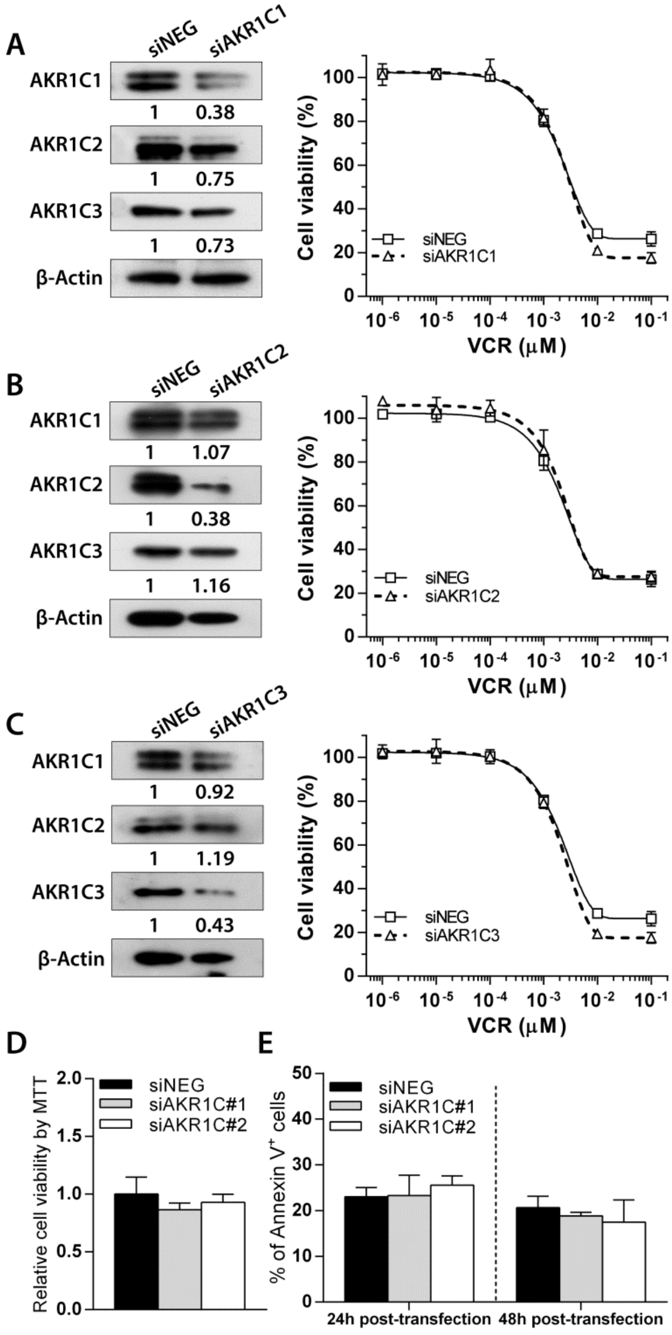

Supplementary Figure S5. Evaluation of the effects exerted by single or combined AKR1C1-3 silencing in CCRF-CEM cells. (**A-C**) After 48h from electroporation with specific siRNAs against AKR1C1 (**A**), AKR1C2 (**B**) and AKR1C3 (**C**) CCRF-CEM cell lysates were analyzed by immunoblotting with specific antibodies, showing the effective and specific gene silencing of each AKR1C1-3 enzymes. Relative densitometric values of bands normalized to β-Actin expression are reported below each protein analyzed (left panels). Response of single AKR1C-silenced CCRF-CEM cells to scalar doses of VCR (48h) is reported and compared to siNEG-transfected control cells (right panels). (**D**) Bar graph showing the relative proliferative capability of AKR1C1-3 transiently silenced CCRF-CEM after 72h from transfection compared to siNEG transfected cells (through MTT assay). (**E**) The same cells have been tested for eventual apoptosis induction at 24h and 48h post-transfection, demonstrating no significant changes by Annexin V staining.

Supplementary Figure S6

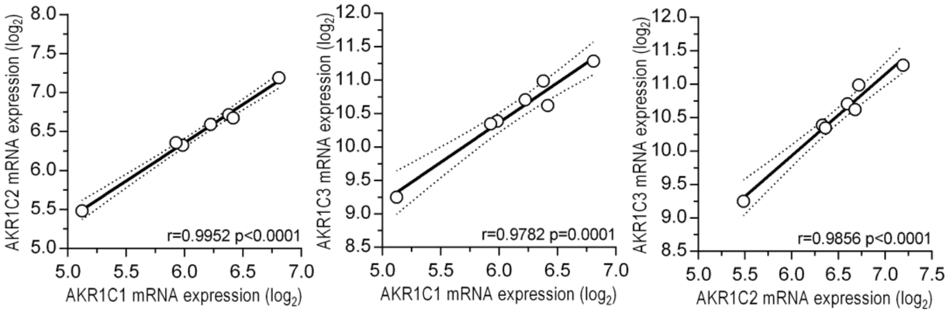

Supplementary Figure S6. Correlation of *AKR1C* isoenzymes expression in T-ALL xenografts. Correlation between the mRNA expression of *AKR1C1 versus AKR1C2* (left panel), *AKR1C1 versus AKR1C3* (middle panel) and *AKR1C2 versus AKR1C3* (right panel) are shown. Pearson coefficient r and relative p value are reported.

REFERENCES

Haferlach T, Kohlmann A, Wieczorek L, Basso G, Kronnie GT, Bene MC, De Vos J, Hernandez JM, Hofmann WK, Mills KI, Gilkes A, Chiaretti S, Shurtleff SA, Kipps TJ, Rassenti LZ, Yeoh AE, Papenhausen PR, Liu WM, Williams PM, Foa R (2010) Clinical utility of microarray-based gene expression profiling in the diagnosis and subclassification of leukemia: report from the International Microarray Innovations in Leukemia Study Group. *J Clin Oncol* **28**(15)**:** 2529-37

Johnson WE, Li C, Rabinovic A (2007) Adjusting batch effects in microarray expression data using empirical Bayes methods. *Biostatistics* **8**(1)**:** 118-27

Milani G, Rebora P, Accordi B, Galla L, Bresolin S, Cazzaniga G, Buldini B, Mura R, Ladogana S, Giraldi E, Conter V, Te Kronnie G, Valsecchi MG, Basso G (2014) Low PKCalpha expression within the MRD-HR stratum defines a new subgroup of childhood T-ALL with very poor outcome. *Oncotarget* **5**(14)**:** 5234-45

Romagnoli R, Baraldi PG, Cruz-Lopez O, Lopez Cara C, Carrion MD, Brancale A, Hamel E, Chen L, Bortolozzi R, Basso G, Viola G (2010) Synthesis and antitumor activity of 1,5-disubstituted 1,2,4-triazoles as cis-restricted combretastatin analogues. *J Med Chem* **53**(10)**:** 4248-58

Schrappe M, Valsecchi MG, Bartram CR, Schrauder A, Panzer-Grumayer R, Moricke A, Parasole R, Zimmermann M, Dworzak M, Buldini B, Reiter A, Basso G, Klingebiel T, Messina C, Ratei R, Cazzaniga G, Koehler R, Locatelli F, Schafer BW, Arico M, Welte K, van Dongen JJ, Gadner H,

Biondi A, Conter V (2011) Late MRD response determines relapse risk overall and in subsets of childhood T-cell ALL: results of the AIEOP-BFM-ALL 2000 study. *Blood* **118**(8)**:** 2077-84
